# Establishing a learning agenda for learning health system implementation and research in Canada

**DOI:** 10.1371/journal.pone.0323499

**Published:** 2025-08-05

**Authors:** Carly Whitmore, Marissa Bird, Shelley Vanderhout

**Affiliations:** 1 School of Nursing - McMaster University, Hamilton, ON, Canada; 2 INTREPID Lab - Centre for Addiction and Mental Health, Toronto, ON, Canada; 3 Learning Health Hub, Mississauga, ON, Canada; 4 Institute for Better Health - Trillium Health Partners, Mississauga, ON, Canada; 5 Institute of Health Policy, Management, and Evaluation - University of Toronto, Toronto, ON, Canada; City University of New York, UNITED STATES OF AMERICA

## Abstract

Health systems in Canada struggle to generate and use knowledge to improve equity-centred quadruple aim measures, resulting in care that is misaligned with local contexts. Learning Health Systems (LHS) offer a solution by aligning real-time evidence, informatics, patient-provider partnerships, and institutional strategies to support continuous improvements in care. Despite their potential, LHS initiatives in Canada remain siloed and lack harmonized leadership, knowledge exchange, and capacity building. To address these gaps, the Learning Health Hub was established to foster collaboration, disseminate best practices, and enhance collective capacity for LHS in Canada. In June 2024, the Learning Health Hub hosted its inaugural virtual symposium. This event brought together partners, researchers, health professionals, system operators, and policymakers from across Canada interested in LHS work. The symposium aimed to map assets and build momentum for larger-scale impact in LHS. Participants engaged in generative activities to define challenges and co-create solutions, resulting in the identification of key learning priorities. Three learning themes were identified: Patient, Caregiver, and Community Partnership; Enabling Environments; and Benchmarking and Evaluation. By advancing these themes, the Learning Health Hub aims to drive meaningful, sustainable change and improve healthcare quality and outcomes.

## Introduction

Health systems across Canada are missing opportunities to generate and use available knowledge to inform and improve equity-centred quadruple aim measures [1,2]. The slow and often fragmented application of evidence to health systems results in care that is outdated and poorly aligned to local factors, including patient population characteristics and organizational culture [2]. An inability to systematically capture and analyze data, as well as generate and use evidence to improve care and care delivery results in a sluggish and unresponsive healthcare system where patients, providers, and communities suffer.

In Learning Health Systems (LHS), real-time evidence, informatics systems, patient-provider partnerships and experiences, and institutional strategies are aligned to support continuous innovation and improvements in care [3]. The aim is to recognize the unique perspectives and priorities of those who receive, provide, fund, and oversee care, as well as address the structures and environmental conditions impacting health system operations [4]. Continuous, rapid, and agile learning cycles in an LHS involve identifying knowledge gaps, applying evidence to create new solutions, exploring and testing them, learning from initial experiences, and iteratively improving the ways that care is organized, delivered, and financed [4]. These learning cycles build evidence for how healthcare systems can deliver patient-centered care and its impact on the social drivers of health and well-being [5].

In Canada, there are a handful of groups and institutions that have recognized the potential for LHS to transform status quo intersections of learning, evidence, and everyday care [3,4]. However, these micro-movements tend to be siloed and specialized in focus, with lessons learned in applying an LHS approach not widely spread [4,5]. As LHS are often envisioned and carried-out by healthcare system leaders, operators, and providers, protected time and resources for learning, implementing change, and knowledge transfer are limited or lacking. Despite best efforts to share innovations in methodology and approach specific to LHS, Canada is lacking the harmonized leadership, knowledge exchange, and capacity building required to support and advance the LHS movement [6]. This scattered approach has underscored an urgent need for the development of a learning infrastructure to ensure progress and equitable access to transformative approaches like LHS.

### Learning health hub.

To cultivate the needed collaboration and integration of LHS work in Canada, the Learning Health Hub was established in fall 2023. Serving as a centralized network for those doing LHS work, the mission of the Learning Health Hub is to foster collaboration and integration of LHS work across Canada, disseminate best practices for LHS, and enhance collective capacity to drive improvements in healthcare quality and outcomes. Partnered with leading healthcare organizations, including Trillium Health Partners’ Institute for Better Health and national Strategy for Patient-Oriented Research (SPOR) Support for People and Patient-Oriented Research and Trials (SUPPORT) units in British Columbia, Alberta, Saskatchewan, Ontario, Quebec, and the Maritimes, academic and research institutions such as the Institute for Health Services and Policy Research, and community groups like the Patient Access Network, the Learning Health Hub is committed to fostering deep, sustained connections between health systems, patients and communities, and researchers working and catalyzing fast-paced, evidence-based change in healthcare.

In June 2024, the Learning Health Hub launched by hosting its inaugural event, a virtual symposium. This virtual symposium convened patient partners, researchers, health professionals, system operators, trainees, and policymakers doing LHS work in Canada with the express purpose of mapping assets, identifying needs and barriers, and building momentum for larger-scale impact. The virtual symposium was co-designed with patient partners and LHS experts to provide a forum to synthesize current knowledge, identify problem areas, challenges, and gaps in LHS implementation and research, assess barriers, and strategize for approaches to solution these challenges, gaps, and barriers. Concurrently, the virtual symposium was used as a platform on which to assemble and map out a network of interested individuals within the Learning Health Hub, keen to move an implementation and research agenda forward.

The purpose of this paper is to document the inaugural LHH symposium as a foundational effort to advance the LHS movement in Canada and to synthesize its outputs into a cohesive learning agenda. Specifically, this paper aims to: 1) map the challenges and opportunities in implementing and sustaining LHS approaches identified and 2) identify core learning priorities for future LHS work. Through this synthesis, this paper seeks to propose strategies to address equity, scalability, and collaboration challenges and catalyze collective efforts to transform Canadian health systems.

## Methods

The two half-day virtual symposium was broadly advertised in established networks using word of mouth, social media (X, LinkedIn), and within professional and patient partner communities such as through Canada-wide SPOR SUPPORT unit email lists. There were no inclusion or exclusion criteria for participation, with all doing, leading, or interested in LHS work welcome to attend. Recognizing that symposium participants were from different regions across Canada and would be at various stages of their LHS work, promotional and reading materials were co-developed with patient partners to be at a grade 8 language level and circulated to maximize productivity in advance of the symposium via email. This included a copy of an applied action framework developed to support the integration of research into health systems [4]. This was intended ensure a common understanding of key concepts and prepare participants to meaningfully engage in co-defining challenges and co-creating approaches to solutions for LHS in Canada.

Participants were encouraged to attend both half-days, which included two keynote presentations as well as interactive, generative small and large group discussions supported by a team of 14 trained facilitators. Generative activities are creative exercises that aim to map participant manifest and latent needs by encouraging ‘out-of-the-box’ thinking by exploring challenges and creating artefacts, representative of alternative future states [7]. In creating these artefacts, participants work together to collaboratively define challenges experienced by individuals in their particular situations before imagining resolutions to those challenges [8]. Generative techniques have previously been used successfully in healthcare service design [9], and healthcare innovation design [7]. The use of generative techniques as the basis for symposium activities was chosen to increase the relevancy, acceptability, and specificity of findings.

### Generation and analysis.

Both half-days of the symposium followed the same structure, which included a brief 30-minute presentation followed by a structured generative activity during the remaining time (see Supplemental File 1 – Virtual Symposium Agenda in S1 Table). The first half-day of the symposium opened with the Learning Health Hub Co-Leads discussing the genesis of the Hub and offering strategic visioning for the ways that it may foster collaboration and integration of LHS work in Canada. This introduction was intended to set the stage for symposium objectives and inspire participants to engage fully with the planned activities. A formal welcome to patients, caregivers, and community members was then offered from two patient partners.

Following this welcome, the first keynote presentation was delivered. This presentation focused on the learning imperative present in health systems and included a review of the international LHS landscape to situate LHS progress in Canada. Applying this content and context, participants were randomized to groups of approximately 10 and tasked with first brainstorming a list of key challenges experienced in their LHS work, and then collaboratively prioritizing these challenges using nominal group techniques [10]. Using a generative activity template, each facilitator presented a high-level description of their priority challenges back to the large group. At the close of the first day, these completed templates were collected, reviewed in full, and thematically analyzed and grouped by the Learning Health Hub Co-Leads, which distilled this comprehensive list of challenges into coherent categories to support activities in the second half of the symposium.

In the second half-day, a recap of day one was provided, including a summary of the themed challenges identified in the first generative activity. Following this, a keynote was given on the complexities and challenges involved in equity-centred LHS work and included practical guidance for considering how equity can be both impacted and prioritized by LHS. Participants then self-selected into small groups specific to one of the thematic challenge categories that were identified from the previous day. Small groups were instructed to apply what they learned from the keynote presentation to identify and elucidate possible approaches to equity-centred solutions for their key challenge. Group facilitators then presented these approaches back to the large group, including what a ‘pie in the sky’ solution would look like, specific issues contributing to the challenge, and a first actionable step that could be taken to address their challenge (see Box 1 for example questions and prompts used by facilitators).

Throughout the second half-day of the symposium, a graphic illustrator was present to capture key insights and visually represent the discussion, creating a dynamic and engaging record of the symposium activities and outcomes. Annotated generative activity templates completed by small group facilitators, notes from symposium leads, and messages contributed to the chat by participants were collected, thematically analyzed, and interpreted to support reflection about the symposium and set a learning agenda to steer future LHS implementation and research.

**Box 1. Questions and prompts.**

Challenge Identification

What is an important challenge that you have encountered or are passionate about in doing LHS work?.

• What are the specific issues contributing to this challenge?
• What are the reasons that this challenge is important?
• Who is impacted by this challenge?
• How is equity implicated and affected by this challenge?

Solution Generation

• What is a potential solution or approach for this challenge?
• What would a ‘pie in the sky’ solution look like?
• What is the first actionable step that needs to be taken to address this challenge?

### Ethical Approval

An ethics review was not sought for this work as the activities conducted during the Learning Health Hub virtual symposium were focused on professional collaboration, knowledge exchange, and the co-creation of ideas rather than on research involving human subjects. However, all participants were informed of the purpose and nature of the symposium, including how the outcomes would be used to inform future work. Symposium attendees provided verbal consensus consent by agreeing to participate in the symposium and engage in generative activities, with the understanding that their contributions would be documented, analyzed, and reported in a manner that respects their confidentiality and anonymity, and collectively agreeing through open discussion and the absence of objections to the use of meeting outcomes.

## Results

Over two half-days, the Learning Health Hub virtual symposium engaged over 130 attendees from across Canada. Representing broad sectors, settings, and roles, symposium participants included researchers, clinicians, health system operators, administrators, and leaders, trainees, and patients, caregivers, and community members who had a range of experience building, implementing, and optimizing LHS.

Following the first day of generative activities, over 25 distinct challenges were collapsed and thematically grouped to five challenge concepts: 1) equity; 2) patient engagement; 3) leadership and decision making; 4) implementation; and 5) measurement, with sub-challenges described in each category (see Supplemental 2 – Summary of Challenges in S1 File). From these challenge categories and following the activity in the second symposium half-day, participants identified a variety of potential solution approaches in their small groups. Based on these challenges and approaches and informed by notes from facilitators and Co-Leads during large group discussion, three learning themes for future implementation and research work were identified. Additionally, and developed from the notes made and questions posed during small generative groupwork, guiding questions for each of the learning themes, posed to guide future implementation and research work, were identified (Table 1).

**Table 1:** Learning theme guiding questions.

Learning Theme	Guiding Questions for Future Implementation and Research
Patient, Caregiver, and Community Partnership	*How can we systematically capture and prioritize diverse data types, including qualitative data of patient, caregiver, and community member experiences in LHS work?* *What strategies and standards can we implement to ensure sustainable and meaningful engagement with equity-deserving groups in health system decision-making processes?* *How can health system priorities be better informed by the experiences and outcomes of people who experience inequities?* *What resources are needed to support accessible, meaningful, and representative engagement and partnership?* *How can we build and sustain relationships with patients, caregivers, and community members that avoid duplication, build on existing community structures, and reach those who are not traditionally engaged?*
Enabling Environments	*How can LHS leadership cultivate trust, integrity, and transparency in decision-making, particularly around priority setting and resource allocation?* *How can we ensure that members of equity-deserving groups, patients, community members, and point-of-care staff are included in decision-making?* *What mechanisms can be implemented to incentivize and enable embedded research, and how should priorities be selected for study?* *What resources, including financial, human, ethical, and regulatory, are necessary to support the implementation of LHS work?* *What strategies can be employed to foster a culture of curiosity, innovation, and learning from failure within health systems?*
Benchmarking and Evaluation	*What tangible examples of LHS can be shared that everyone can understand and see themselves in, and how can these be scaled across diverse contexts?* *How can LHS demonstrate leadership and share best practices with one another?* *How can we foster an environment where learning is seen as improvement rather than judgment, and how can we support the de-implementation of ineffective practices?* *What combination of evaluation methods (e.g., quantitative, realist, process, qualitative) should be used to provide a robust understanding of high-quality LHS performance?* *How can we ensure that health systems take the time to understand what doesn’t work and why, and what steps can be taken to learn from and adapt based on these failures?*

### Learning theme 1: Patient, caregiver, and community partnership

**Fig 1:**
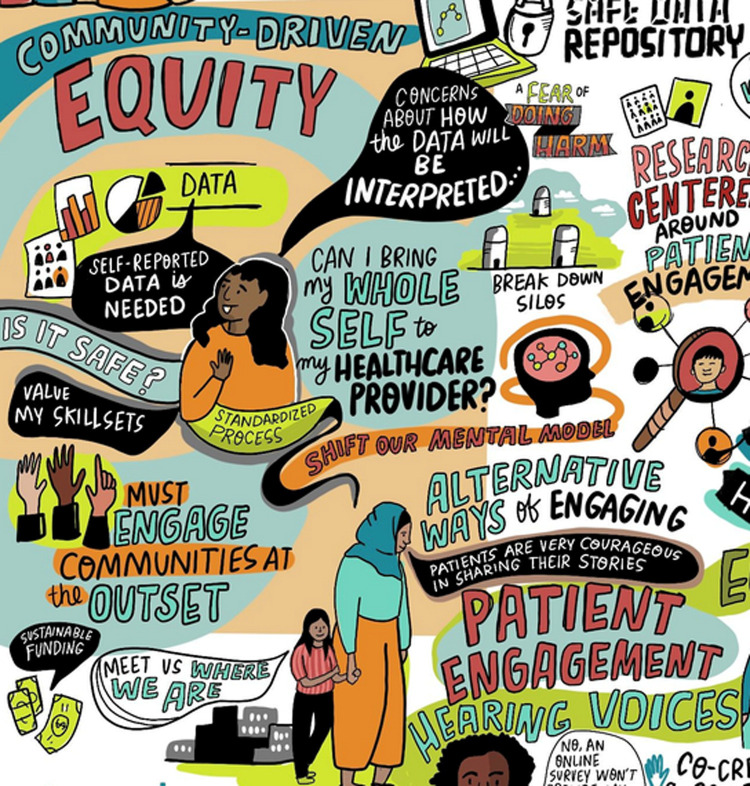
Partnership.

Establishing sustainable, trusting, and mutually beneficial relationships with patients, caregivers, and community members in LHS work was identified as essential for aligning LHS priorities, engagement processes, and knowledge sharing approaches with community needs and priorities. When engaging with patients, caregivers, and communities, symposium participants also highlighted the importance of incorporating diverse types of data, removing barriers to engagement, and ensuring partnership with equity-deserving groups. This discussion identified a strong appetite on behalf of patient and community partners to be invited to dialogue with LHS leaders about needs and expectations around how partnerships should occur. In the absence of these discussions, health system partners are left poorly prepared to meaningfully, sustainably, and safely partner with patients, caregivers, and communities, risking tokenism and worsening historically poor relationships between health systems and equity-deserving groups (Fig 1).

### Learning theme 2: Enabling environments

**Fig 2:**
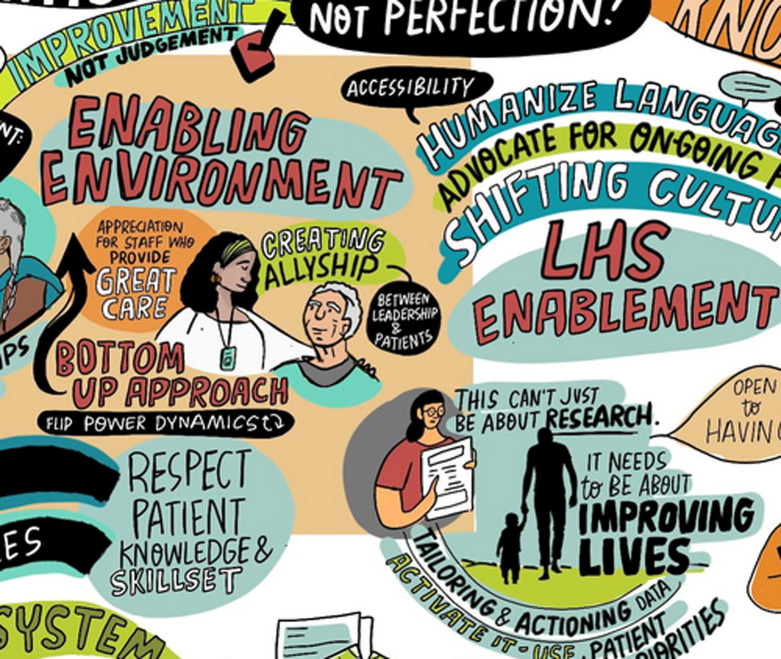
LHS enablement.

Creating environments where learning thrives involves fostering leadership, embedding research processes, and cultivating a culture of curiosity. To do this, it was identified that trust, transparency, and inclusion in decision-making, alongside dedicated resources and synergistic governance was required. These elements together hold the potential to support the implementation, maturity, and sustainability of LHS. Participants identified the importance of building a LHS culture that allows healthcare providers, patients, and leaders to clearly understand their unique roles in contributing to the day-to-day operation and strategic direction of LHS. Emphasizing continuous feedback loops, leveraging diverse perspectives, and ensuring that every stakeholder feels valued and empowered are crucial for creating an adaptable and resilient learning health system that can effectively respond to evolving healthcare needs and challenges (Fig 2).

### Learning theme 3: Benchmarking and evaluation

**Fig 3:**
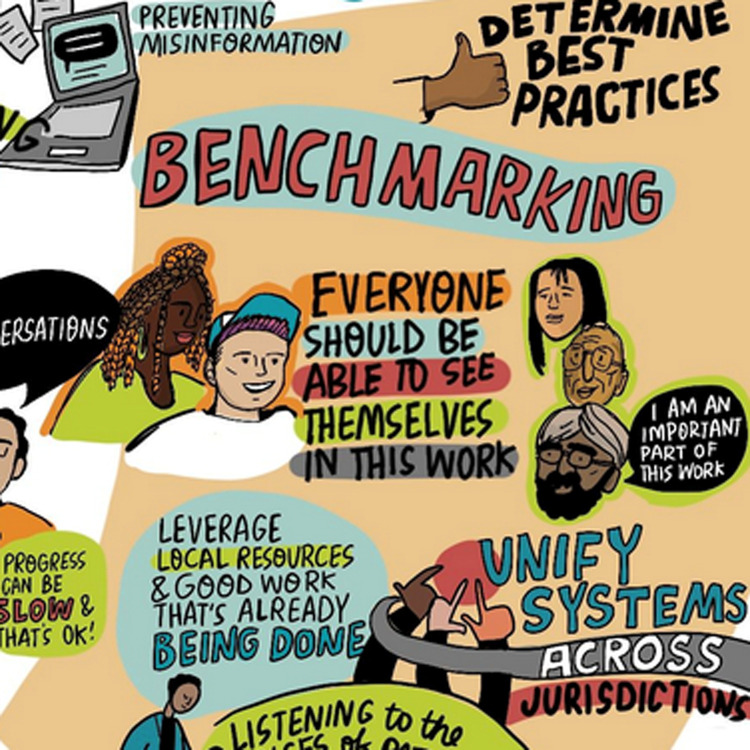
Benchmarking.

Benchmarking and evaluation in LHS focus on how data is used, ensuring a shared understanding of goals, and co-defining the methods and approaches that are most appropriate across settings and evaluation types. Developing a common language around and about LHS as well as sharing tangible examples, promoting best practices, and using a mix of evaluation methods ensure continuous improvement and adaptation within health systems. This collaborative approach helps to identify gaps, recognize achievements, and drive innovation, ultimately enhancing the quality and efficiency of healthcare delivery. By fostering an environment of transparency and mutual learning, stakeholders can more effectively contribute to and benefit from the LHS, creating a more responsive and equitable healthcare system (Fig 3).

These three themes and the questions posed are depicted in the generated graphic illustration (Supplemental File 3 – Graphic Illustration in S1 Fig).

## Discussion

The Learning Health Hub and its inaugural virtual symposium represent a pioneering effort in Canada to establish a national LHS platform and form a foundation for unified learning and innovation in healthcare. Developed through generative activities, the three identified learning themes – Patient and Community Partnership, Enabling Environments, and Benchmarking and Evaluation – underscore the critical elements needed to drive future implementation and research efforts in LHS work. While these three themes are distinct and address different aspects of LHS, each reinforces and supports the others, creating a synergistic framework for advancing LHS work. This interconnectedness highlights a need to explore these learning themes collectively and collaboratively to ensure we make progress on inclusive engagement, foster a culture of continuous learning, and provide actionable insights for improvement. Aligned to these three learning themes and illustrating their synergies, the following section will discuss the importance of equity, sustainability and scalability, and collaboration in LHS work.

### Equity

Embedded throughout the virtual symposium as a core focus, a critical area for future exploration is the systematic integration of equity into all aspects of LHS work. This includes ensuring that patient engagement, leadership and decision-making, and evaluation practices are designed to not only address disparities in healthcare outcomes, but also but also to promote fairness and inclusivity across all levels and processes of the health system. In setting up the LHS for success in promoting equity across all aspects, it is important to recognize and attend to systemic failures of the health system over time that have brought about or widened health inequities and poor health outcomes for socially disadvantaged groups. Evidence of these disparities persist in examples such as the disproportionately high burden of chronic conditions in Indigenous Canadians [11], and in the inequitable morbidity and mortality rates seen globally in infectious disease epidemiology, including in the recent COVID-19 pandemic [12]. It is critically important for LHS practitioners to learn from both historical and current ethical failures in the health system such that history not repeat itself, and to dynamically respond to population health needs as they arise. Additionally, to restore trust and promote meaningful partnership with patients and caregivers within LHS, transparency, going to where people are, nurturing creativity, and promoting thriving, is imperative [13,14].

Drawing on the broader literature on health equity and structural and social determinants of health can provide a deeper understanding of how to address disparities in health and care outcomes, inform strategies for this integration into LHS implementation and research, and lead to more effective health interventions. This includes the intentional infusion of core practices in the pursuit of equity, including establishing principle, measuring for equity, leading from lived experience, co-producing, redistributing power, practicing a growth mindset, and engaging beyond the healthcare system [15], and the use of structures such as the PETAL framework (Prioritize health equity; Engage the community; Target health disparities; Act on the data; and Learn and improve) [16].

### Sustainability and scalability

Another important area of focus in moving this learning agenda forward is the sustainability and scalability of LHS work. Investigating the factors that contribute to the long-term success and adaptability of LHS initiatives can help ensure that successful models are shared and replicated in various contexts. This involves understanding the elements that support the maturation and sustained impact of LHS, including robust data systems, resource allocations, and stakeholder engagement. Literature from implementation science and system transformation can offer valuable insights into how to build and maintain sustainable and scalable LHS models. For example, research on sustainability frameworks and maturity models can guide the development of practices that ensure the longevity and expansion of successful LHS initiatives.

Foundational research such as the Dynamic Sustainability Framework offers an opportunity to identify the stable and foundational ‘core’ elements, as well as peripheral elements that can and should be tailored to fit local context and scale LHS [17]. These may include core functionalities, analytics, use of evidence, co-design and implementation, evaluation, change management or governance structures, data sharing, knowledge sharing, training and capacity building, equity, and sustainability [4,18,19]. In addition, attention to the implementation setting such as organizational characteristics (culture, structure, climate, resources [17]) and their fit with LHS characteristics, LHS practitioners may enhance sustainability and scalability of LHS over time.

### Cross-sectoral collaboration

Lastly, there are significant opportunities to explore the essential role of cross-sectoral collaboration in advancing LHS work. While existing efforts in LHS are often siloed and focused on specific areas, promoting partnerships across different sectors and settings can lead to more integrated and effective healthcare solutions. However, to learn at a systems scale, the distance between network members must be more equal [20], as these innovation systems facilitate learning through interactive processes that cross organizational boundaries while leveraging diverse sources of knowledge, experience, and capability [21]. This systems-scale learning harnesses collective wisdom defined by the capacity of the networks to enable shared learning and cohesive problem-solving [20]. Literature on collaborative governance and intersectoral partnerships can shed light on effective strategies for fostering such collaboration, highlighting the benefits of a unified approach to health system improvement. However, this requires an investment in building human capital, including developing leadership, change management, and agentic competencies [6]. Additionally, there is a need to ensure effective knowledge management between sectors in this collaboration, where a balance between tacit knowledge and explicit knowledge is maintained, as practical, on-the-ground insights need to complement formal, codified information [22]. By embracing cross-sectoral collaboration, LHS can leverage diverse perspectives and resources to address complex health challenges more comprehensively, ultimately leading to more integrated, effective, and equitable healthcare solutions.

While the implementation, maturity, and sustainability of LHS remain nascent, there is an opportunity to learn from extant literature to advance these learning priorities. The Learning Heath Hub and the completed virtual symposium has served as a catalyst for these needed national collaborative efforts, capable of driving meaningful, sustainable change. By forging new relationships across sectors and systems, strengthening existing partnerships, and building capacity in embedded research, this learning agenda provides the scaffolding required to enable this learning network to self-organize [20].

## Conclusion

Laying the groundwork for the development of a learning agenda, symposium findings have identified key questions to guide future research, policy, and practice efforts in healthcare systems across Canada. By harnessing national momentum and promoting diverse partnerships, the Learning Health Hub will support the transformational and agile uptake of evidence, build capacity, and foster a culture of learning. This includes a focus on the mechanisms and processes of care integration and the ways that care delivery can capture and address structural and social determinants of health, support cross-sectoral care delivery, and leverage existing infrastructure.

## Supporting information

S1 Table Virtual Symposium Agenda. (DOCX)

S1 File Summary of Challenges. (DOCX)

S1 Fig Graphic illustration. (JPG)
