# Supplementary material for: Establishing a learning agenda for learning health system implementation and research in Canada
Source: PLoS One. 2025 Aug 5;20(8):e0323499. doi: 10.1371/journal.pone.0323499 (PMC12324668; doi:10.1371/journal.pone.0323499)
Supplement: S1 Table — (DOCX) [file pone.0323499.s001.docx]

Supplemental File 1: Learning Health Hub Virtual Symposium Agenda

| **Day One**  **June 6, 2024: 1200 – 1500hrs EST** | | |
| --- | --- | --- |
| **Time** | **Focus** | **Notes** |
| 1200 -1230 | **Welcome**  **Vision for the Learning Health Hub network**  **Symposium Overview** | *Team introductions*  *Land acknowledgement*  *Patient partner welcome*  *Symposium impact goals*  *Review agenda* |
| 1230 – 1300 | **Keynote Presentation 1**: Building Momentum for Learning Health Systems in Canada | *Speakers:*  *Robert Reid MD PhD Chief Scientist Institute for Better Health, Trillium Health Partners*  *Sarah Greene MPH Senior Advisor,  National Academy of Medicine* |
| 1300 - 1430 | Activity 1:  Co-Envisioning Challenges of Learning Health System Work in Canada | *Template:*   - *Idea generation* - *Idea prioritization* - *Guided challenge discussion* |
| 1430 - 1445 | Share Back | *Large group* |
| 1445 -1500 | Group Reflections  Looking Ahead to Day 2 | *Overview Day 2 agenda* |

| **Day Two**  **June 7, 2024: 1200 – 1500hrs EST** | | |
| --- | --- | --- |
| **Time** | **Focus** | **Notes** |
| 1200 -1215 | **Recap from Day 1**  **Presentation of Challenge Themes**  **Introduce Graphic Illustrator** | *Participants self-select challenge group of interest* |
| 1215 – 1245 | **Keynote Presentation 2**: Complexities and Challenges in  Equity-Centred LHS Work | *Speakers:*  *Ibukun Abejirinde MD PhD Scientist Institute for Better Health, Trillium Health Partners*  *Brianne Wood PhD Associate Scientist Thunder Bay Regional Health Research Institute and the Northern Ontario School of Medicine* |
| 1245 - 1330 | Activity 2:  Approaches to Solution Generation | *Template:*   - *‘Pie in the sky’* - *Actionable steps* - *Equity focus*   *Graphic artist circulates in small groups* |
| 1330 - 1445 | Share Back | *Large group*  *Spotlight on graphic illustration* |
| 1445 -1500 | Co-Envisioning a Learning Health System Network in Canada | *Where are we headed?*  *Network building* |
