# Supplementary material for: Establishing a learning agenda for learning health system implementation and research in Canada
Source: PLoS One. 2025 Aug 5;20(8):e0323499. doi: 10.1371/journal.pone.0323499 (PMC12324668; doi:10.1371/journal.pone.0323499)
Supplement: S1 File — (DOCX) [file pone.0323499.s002.docx]

**Supplemental File 2 – Summary of Challenges**

**Themes from LHH challenges**

1. Equity
   1. Which types of data are prioritized?
      1. Different ways of learning and knowing
      2. Data hierarchy – we have access to so much data, but SDOH, equity, qualitative data, patient and provider experience not meaningfully and systematically captured, prioritized
   2. Equity-deserving groups often not engaged in ways that are sustainable, equitable, and in meaningful conversations (e.g., about decision making)
   3. Health system priorities need to be informed by the experiences and outcomes of people facing inequities
   4. Need to define what equity looks like in different contexts
2. Patient engagement
   1. Disconnect between needs of patients and communities and health system priorities
   2. Need financial and human resources to support accessible, non-tokenistic, representative patient engagement
   3. Need to better define who is and is not being reached – patient engagement isn’t just about health system users
   4. Need common space for dialogue that minimizes power imbalances, engages equity deserving groups, uses common language, minimizes barriers
3. Leadership and decision making towards a LHS-enabling environment
   1. Trust, integrity, and transparency are needed in decision making, especially around priority setting and allocation of resources
   2. Members of equity-deserving groups and patients/community members, front line staff need to be included
   3. How is embedded research incentivized, enabled, and which problems are chosen?
4. Implementation
   1. Implementation resources to support LHS work (not off the side of the desk) - financial to support time, human resources to support capacity and skill building
   2. Privacy, ethics, regulations, data sharing must work synergistically and orient around LHS processes
   3. Culture of curiosity, innovation, learning from failure, readiness for change
5. Benchmarking, evaluation, measurement of best practices
   1. Shared understanding and language about what a LHS is and looks like
      1. What are tangible examples that everyone can understand and “see themselves in?”
   2. How do these scale and look different across diverse contexts with varying resources
   3. How do LHS demonstrate leadership and share best practices with one another?

Themes from LHH Solutions

**Solutions Notes**

**Core themes**

1. Embracing failure and cultivating learning and curiosity
   1. Slowness is okay
   2. Take the time to understand what doesn’t work and why, de-implement and re-allocate resources
   3. Improvement, not judgment – learning is not punitive
2. Building sustained relationships with community partners
   1. Health systems need dedicated financial and human resources for this to promote accessibility and sustainability, reduce barriers to engagement
   2. Developing engagement with community groups by looking outside of healthcare to avoid duplication and build on existing structures
   3. Cross-sectoral, diverse representation, especially from people without attachment to the system
   4. Institutions prioritize rapid processes, but relationships are slow
   5. REBs need to check that engagement is happening and equity is addressed
3. Leadership and decision making
   1. Keep re-evaluating who is making decisions and who’s at the table
   2. Researchers and leadership need to create allyship for patient engagement beyond the project and toward the system level
   3. Health systems could consider a LHS chief officer who is a knowledge broker, boundary spanner who could express value of LHS to other senior leaders by “speaking their language”
   4. Transparent and accountable governance structures, monitoring, evaluation, and reporting mechanisms
4. Culture shift and establishing meaning
   1. Everyone needs to feel supported and comfortable to contribute; see themselves in the work where an opportunity for improvement would be recognized, meaningful, and exciting to all
   2. Front line staff need to be a part of the process, need to understand the goal of better care, and envision what their role is in achieving it
