# Supplementary figures and images for: Establishing a learning agenda for learning health system implementation and research in Canada
Source: PLoS One. 2025 Aug 5;20(8):e0323499. doi: 10.1371/journal.pone.0323499 (PMC12324668; doi:10.1371/journal.pone.0323499)

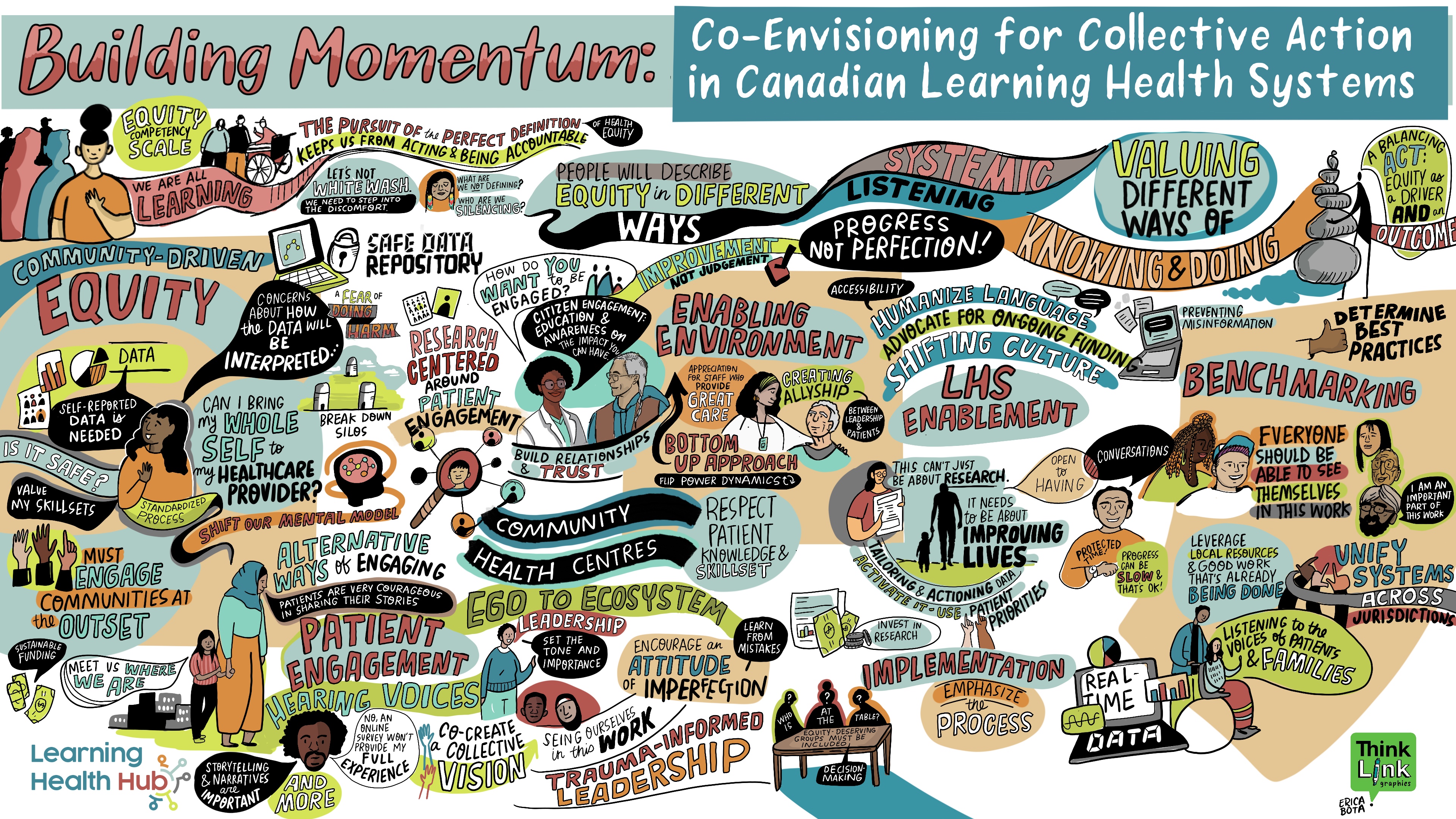

Supplement: S1 Fig — (JPG) [file pone.0323499.s003.jpg]
